# Supplementary material for: Modeling glioblastoma heterogeneity as a dynamic network of cell states
Source: Mol Syst Biol. 2021 Sep 16;17(9):e10105. doi: 10.15252/msb.202010105 (PMC8444284; doi:10.15252/msb.202010105)
Supplement: Supplementary file 5 — Source Data for Figure 3 [file MSB-17-e10105-s001.zip › Figure3A_sourcedata/GSEA_3065/nosigdb_state2.GseaPreranked.1622718419215/DOROSZKO_U3180MG_CLUSTER_4.html]

Details for gene set DOROSZKO\_U3180MG\_CLUSTER\_4[GSEA]

|  || Dataset | state2 |
| Phenotype | NoPhenotypeAvailable |
| Upregulated in class | na\_pos |
| GeneSet | DOROSZKO\_U3180MG\_CLUSTER\_4 |
| Enrichment Score (ES) | 0.69096935 |
| Normalized Enrichment Score (NES) | 2.4449205 |
| Nominal p-value | 0.0 |
| FDR q-value | 0.0 |
| FWER p-Value | 0.0 |
Table: GSEA Results Summary

  

Fig 1: Enrichment plot: DOROSZKO\_U3180MG\_CLUSTER\_4      
 Profile of the Running ES Score & Positions of GeneSet Members on the Rank Ordered List

  

| PROBE | GENE SYMBOL | GENE\_TITLE | RANK IN GENE LIST | RANK METRIC SCORE | RUNNING ES | CORE ENRICHMENT || 1 | UBE2S |  |  | 19 | 0.574 | 0.0242 | Yes |
| 2 | PTTG1 |  |  | 63 | 0.439 | 0.0396 | Yes |
| 3 | CCNB1 |  |  | 68 | 0.420 | 0.0583 | Yes |
| 4 | NUCKS1 |  |  | 78 | 0.404 | 0.0758 | Yes |
| 5 | CENPE |  |  | 105 | 0.372 | 0.0900 | Yes |
| 6 | ANLN |  |  | 111 | 0.358 | 0.1058 | Yes |
| 7 | CDKN2C |  |  | 123 | 0.341 | 0.1202 | Yes |
| 8 | KIF11 |  |  | 129 | 0.335 | 0.1350 | Yes |
| 9 | CEP55 |  |  | 158 | 0.306 | 0.1459 | Yes |
| 10 | ECT2 |  |  | 171 | 0.297 | 0.1582 | Yes |
| 11 | MZT1 |  |  | 174 | 0.295 | 0.1715 | Yes |
| 12 | CCNB2 |  |  | 190 | 0.286 | 0.1829 | Yes |
| 13 | CDC20 |  |  | 193 | 0.284 | 0.1957 | Yes |
| 14 | KIF20B |  |  | 208 | 0.278 | 0.2069 | Yes |
| 15 | JPT1 |  |  | 236 | 0.260 | 0.2159 | Yes |
| 16 | CENPA |  |  | 238 | 0.259 | 0.2276 | Yes |
| 17 | ILF2 |  |  | 240 | 0.258 | 0.2392 | Yes |
| 18 | VBP1 |  |  | 245 | 0.254 | 0.2504 | Yes |
| 19 | PPP1CC |  |  | 246 | 0.253 | 0.2620 | Yes |
| 20 | PRR11 |  |  | 285 | 0.238 | 0.2688 | Yes |
| 21 | MAZ |  |  | 290 | 0.236 | 0.2791 | Yes |
| 22 | ITGAE |  |  | 292 | 0.236 | 0.2898 | Yes |
| 23 | KIF14 |  |  | 302 | 0.231 | 0.2994 | Yes |
| 24 | BUB1 |  |  | 335 | 0.222 | 0.3060 | Yes |
| 25 | HMMR |  |  | 358 | 0.215 | 0.3135 | Yes |
| 26 | PLK1 |  |  | 364 | 0.213 | 0.3227 | Yes |
| 27 | TXNDC17 |  |  | 365 | 0.213 | 0.3324 | Yes |
| 28 | DLGAP5 |  |  | 371 | 0.211 | 0.3415 | Yes |
| 29 | AURKA |  |  | 375 | 0.210 | 0.3508 | Yes |
| 30 | COPRS |  |  | 377 | 0.209 | 0.3602 | Yes |
| 31 | GLRX5 |  |  | 386 | 0.207 | 0.3688 | Yes |
| 32 | HNRNPM |  |  | 412 | 0.198 | 0.3752 | Yes |
| 33 | KIF18A |  |  | 419 | 0.195 | 0.3834 | Yes |
| 34 | SKA2 |  |  | 421 | 0.195 | 0.3922 | Yes |
| 35 | IFT22 |  |  | 424 | 0.194 | 0.4009 | Yes |
| 36 | NCAPD2 |  |  | 434 | 0.193 | 0.4087 | Yes |
| 37 | TROAP |  |  | 435 | 0.193 | 0.4175 | Yes |
| 38 | SPAG5 |  |  | 439 | 0.192 | 0.4260 | Yes |
| 39 | CALM3 |  |  | 446 | 0.191 | 0.4340 | Yes |
| 40 | ANP32A |  |  | 457 | 0.189 | 0.4416 | Yes |
| 41 | SPDL1 |  |  | 459 | 0.189 | 0.4501 | Yes |
| 42 | DCP2 |  |  | 474 | 0.184 | 0.4570 | Yes |
| 43 | KNSTRN |  |  | 483 | 0.183 | 0.4645 | Yes |
| 44 | NDUFAF3 |  |  | 489 | 0.182 | 0.4723 | Yes |
| 45 | CDKN2D |  |  | 499 | 0.182 | 0.4797 | Yes |
| 46 | SYNCRIP |  |  | 503 | 0.180 | 0.4875 | Yes |
| 47 | HNRNPH3 |  |  | 504 | 0.180 | 0.4958 | Yes |
| 48 | LDHA |  |  | 506 | 0.179 | 0.5038 | Yes |
| 49 | CBX1 |  |  | 509 | 0.179 | 0.5118 | Yes |
| 50 | CDC25B |  |  | 519 | 0.178 | 0.5190 | Yes |
| 51 | KHDRBS1 |  |  | 548 | 0.173 | 0.5238 | Yes |
| 52 | HDGF |  |  | 570 | 0.168 | 0.5292 | Yes |
| 53 | CKAP5 |  |  | 596 | 0.164 | 0.5340 | Yes |
| 54 | CCT8 |  |  | 620 | 0.160 | 0.5389 | Yes |
| 55 | BZW1 |  |  | 628 | 0.159 | 0.5454 | Yes |
| 56 | TPGS2 |  |  | 632 | 0.158 | 0.5523 | Yes |
| 57 | CDCA8 |  |  | 634 | 0.158 | 0.5594 | Yes |
| 58 | SRRT |  |  | 657 | 0.155 | 0.5641 | Yes |
| 59 | GTF2A2 |  |  | 662 | 0.155 | 0.5707 | Yes |
| 60 | FAM120AOS |  |  | 736 | 0.146 | 0.5695 | Yes |
| 61 | NUDCD2 |  |  | 751 | 0.143 | 0.5746 | Yes |
| 62 | FAM83D |  |  | 759 | 0.143 | 0.5803 | Yes |
| 63 | SSNA1 |  |  | 763 | 0.142 | 0.5865 | Yes |
| 64 | SEPHS1 |  |  | 771 | 0.142 | 0.5922 | Yes |
| 65 | STIP1 |  |  | 794 | 0.139 | 0.5962 | Yes |
| 66 | PSMA4 |  |  | 797 | 0.138 | 0.6023 | Yes |
| 67 | NEK2 |  |  | 805 | 0.138 | 0.6078 | Yes |
| 68 | TTK |  |  | 831 | 0.135 | 0.6113 | Yes |
| 69 | UBALD2 |  |  | 843 | 0.133 | 0.6162 | Yes |
| 70 | RHNO1 |  |  | 857 | 0.132 | 0.6208 | Yes |
| 71 | MRPL47 |  |  | 891 | 0.129 | 0.6231 | Yes |
| 72 | GDI2 |  |  | 897 | 0.128 | 0.6284 | Yes |
| 73 | NUP37 |  |  | 900 | 0.128 | 0.6341 | Yes |
| 74 | TMEM160 |  |  | 913 | 0.126 | 0.6386 | Yes |
| 75 | RBM42 |  |  | 982 | 0.121 | 0.6368 | Yes |
| 76 | CCT7 |  |  | 1029 | 0.117 | 0.6372 | Yes |
| 77 | EIF4E |  |  | 1038 | 0.116 | 0.6416 | Yes |
| 78 | SAPCD2 |  |  | 1064 | 0.114 | 0.6441 | Yes |
| 79 | KIF20A |  |  | 1094 | 0.112 | 0.6461 | Yes |
| 80 | TMEM256 |  |  | 1105 | 0.111 | 0.6501 | Yes |
| 81 | DAZAP1 |  |  | 1151 | 0.108 | 0.6502 | Yes |
| 82 | RANGAP1 |  |  | 1157 | 0.107 | 0.6545 | Yes |
| 83 | RBMX |  |  | 1186 | 0.105 | 0.6563 | Yes |
| 84 | ARPP19 |  |  | 1205 | 0.104 | 0.6592 | Yes |
| 85 | CDK2AP2 |  |  | 1213 | 0.104 | 0.6631 | Yes |
| 86 | NDUFA2 |  |  | 1244 | 0.102 | 0.6645 | Yes |
| 87 | HP1BP3 |  |  | 1249 | 0.102 | 0.6688 | Yes |
| 88 | GAS2L3 |  |  | 1326 | 0.096 | 0.6650 | Yes |
| 89 | TMSB10 |  |  | 1358 | 0.094 | 0.6659 | Yes |
| 90 | GSPT1 |  |  | 1377 | 0.093 | 0.6683 | Yes |
| 91 | DCTN3 |  |  | 1384 | 0.093 | 0.6719 | Yes |
| 92 | ECI2 |  |  | 1422 | 0.091 | 0.6720 | Yes |
| 93 | RUVBL1 |  |  | 1426 | 0.091 | 0.6759 | Yes |
| 94 | RNF5 |  |  | 1473 | 0.088 | 0.6749 | Yes |
| 95 | RAB11A |  |  | 1484 | 0.087 | 0.6778 | Yes |
| 96 | CCDC18 |  |  | 1491 | 0.087 | 0.6812 | Yes |
| 97 | ODF2 |  |  | 1512 | 0.086 | 0.6829 | Yes |
| 98 | USP13 |  |  | 1529 | 0.085 | 0.6851 | Yes |
| 99 | CFAP36 |  |  | 1531 | 0.085 | 0.6888 | Yes |
| 100 | MORF4L2 |  |  | 1548 | 0.084 | 0.6910 | Yes |
| 101 | KATNA1 |  |  | 1711 | 0.076 | 0.6770 | No |
| 102 | REEP4 |  |  | 1734 | 0.076 | 0.6781 | No |
| 103 | TIMM9 |  |  | 1765 | 0.074 | 0.6782 | No |
| 104 | RNF26 |  |  | 1787 | 0.073 | 0.6793 | No |
| 105 | CDC25C |  |  | 1820 | 0.072 | 0.6791 | No |
| 106 | ADI1 |  |  | 1839 | 0.071 | 0.6804 | No |
| 107 | ETV4 |  |  | 1840 | 0.071 | 0.6836 | No |
| 108 | MDH1 |  |  | 1845 | 0.070 | 0.6864 | No |
| 109 | EIF4G2 |  |  | 1887 | 0.069 | 0.6851 | No |
| 110 | BORA |  |  | 1911 | 0.068 | 0.6857 | No |
| 111 | ASF1A |  |  | 2045 | 0.063 | 0.6743 | No |
| 112 | PAIP1 |  |  | 2060 | 0.062 | 0.6756 | No |
| 113 | AIMP2 |  |  | 2087 | 0.061 | 0.6756 | No |
| 114 | RUVBL2 |  |  | 2141 | 0.060 | 0.6726 | No |
| 115 | IMMP1L |  |  | 2152 | 0.059 | 0.6743 | No |
| 116 | EMC9 |  |  | 2216 | 0.057 | 0.6700 | No |
| 117 | PRMT5 |  |  | 2240 | 0.056 | 0.6701 | No |
| 118 | FUBP1 |  |  | 2296 | 0.054 | 0.6666 | No |
| 119 | SPA17 |  |  | 2377 | 0.051 | 0.6604 | No |
| 120 | G2E3 |  |  | 2405 | 0.051 | 0.6598 | No |
| 121 | ARL6IP1 |  |  | 2421 | 0.050 | 0.6604 | No |
| 122 | EEF1AKMT2 |  |  | 2468 | 0.049 | 0.6577 | No |
| 123 | COX20 |  |  | 2606 | 0.044 | 0.6449 | No |
| 124 | AC026401.3 |  |  | 2688 | 0.042 | 0.6381 | No |
| 125 | RANGRF |  |  | 2833 | 0.038 | 0.6243 | No |
| 126 | CRAT |  |  | 2856 | 0.038 | 0.6237 | No |
| 127 | NUDT11 |  |  | 2995 | 0.034 | 0.6103 | No |
| 128 | EZR |  |  | 3039 | 0.033 | 0.6072 | No |
| 129 | SORD |  |  | 3056 | 0.033 | 0.6070 | No |
| 130 | SDCBP |  |  | 3058 | 0.033 | 0.6083 | No |
| 131 | LMO7 |  |  | 3078 | 0.032 | 0.6078 | No |
| 132 | HNRNPA1P48 |  |  | 3098 | 0.032 | 0.6072 | No |
| 133 | TNFAIP8L1 |  |  | 3154 | 0.031 | 0.6026 | No |
| 134 | SEC22C |  |  | 3388 | 0.026 | 0.5787 | No |
| 135 | PPP2R5C |  |  | 3456 | 0.025 | 0.5726 | No |
| 136 | CEP70 |  |  | 3480 | 0.024 | 0.5712 | No |
| 137 | NDE1 |  |  | 3764 | 0.019 | 0.5416 | No |
| 138 | SNAPC1 |  |  | 3813 | 0.019 | 0.5373 | No |
| 139 | RAB13 |  |  | 3928 | 0.017 | 0.5257 | No |
| 140 | CIB1 |  |  | 4002 | 0.015 | 0.5185 | No |
| 141 | PSME2 |  |  | 4036 | 0.015 | 0.5157 | No |
| 142 | TMEM19 |  |  | 4969 | 0.000 | 0.4152 | No |
| 143 | DDAH2 |  |  | 5011 | -0.000 | 0.4107 | No |
| 144 | MAP1LC3B |  |  | 5020 | -0.000 | 0.4099 | No |
| 145 | GPSM2 |  |  | 5127 | -0.002 | 0.3986 | No |
| 146 | PRR13 |  |  | 5446 | -0.008 | 0.3646 | No |
| 147 | HSD17B11 |  |  | 5658 | -0.011 | 0.3423 | No |
| 148 | AAMDC |  |  | 5689 | -0.011 | 0.3396 | No |
| 149 | TXNDC12 |  |  | 5696 | -0.011 | 0.3395 | No |
| 150 | PIF1 |  |  | 5869 | -0.014 | 0.3216 | No |
| 151 | MAP4K4 |  |  | 5903 | -0.015 | 0.3187 | No |
| 152 | PCBD2 |  |  | 5921 | -0.015 | 0.3176 | No |
| 153 | PPP1R35 |  |  | 6058 | -0.017 | 0.3037 | No |
| 154 | IDH1 |  |  | 6208 | -0.020 | 0.2885 | No |
| 155 | KIAA0586 |  |  | 6410 | -0.024 | 0.2679 | No |
| 156 | MEX3A |  |  | 6667 | -0.029 | 0.2416 | No |
| 157 | TMED5 |  |  | 6753 | -0.031 | 0.2339 | No |
| 158 | SMIM14 |  |  | 6812 | -0.032 | 0.2291 | No |
| 159 | LBR |  |  | 7064 | -0.038 | 0.2037 | No |
| 160 | ACTN1 |  |  | 7366 | -0.046 | 0.1734 | No |
| 161 | SUN2 |  |  | 8189 | -0.077 | 0.0882 | No |
| 162 | SUGP2 |  |  | 8244 | -0.079 | 0.0860 | No |
| 163 | HSPA13 |  |  | 8415 | -0.090 | 0.0718 | No |
| 164 | ZNF106 |  |  | 8484 | -0.094 | 0.0688 | No |
| 165 | PDGFRA |  |  | 8503 | -0.096 | 0.0712 | No |
| 166 | CNIH4 |  |  | 8611 | -0.104 | 0.0644 | No |
| 167 | LGALS1 |  |  | 8680 | -0.110 | 0.0621 | No |
| 168 | FKBP2 |  |  | 8892 | -0.134 | 0.0455 | No |
| 169 | EMP3 |  |  | 8918 | -0.137 | 0.0490 | No |
| 170 | PLIN3 |  |  | 9043 | -0.157 | 0.0428 | No |
Table: GSEA details [plain text format]

  

Fig 2: DOROSZKO\_U3180MG\_CLUSTER\_4: Random ES distribution      
 Gene set null distribution of ES for **DOROSZKO\_U3180MG\_CLUSTER\_4**
